# Comprehensive review of pulmonary embolism imaging: past, present and future innovations in computed tomography (CT) and other diagnostic techniques

**DOI:** 10.1007/s11604-025-01811-8

**Published:** 2025-06-28

**Authors:** Sonia Triggiani, Giuseppe Pellegrino, Sveva Mortellaro, Alessandra Bubba, Carolina Lanza, Serena Carriero, Pierpaolo Biondetti, Salvatore Alessio Angileri, Roberta Fusco, Vincenza Granata, Gianpaolo Carrafiello

**Affiliations:** 1 Postgraduation School in Radiodiagnostics, University of Milan, 20122 Milan, Italy; 2 Radiology Department, Fondazione IRCCS Cà Granda, Policlinico di Milano Ospedale Maggiore, 20122 Milan, Italy; 3 Division of Radiology, Istituto Nazionale per lo studio e la cura dei Tumori “Fondazione G. Pascale”, Naples, Italy

**Keywords:** Pulmonary embolism (EP), Computed tomographic pulmonary angiography (CTPA), Dual-energy computed tomography (DECT), Photon-counting detector CT (PCD-CT), Artificial intelligence (AI), Computer-aid detection (CAD)

## Abstract

Pulmonary embolism (PE) remains a critical condition that demands rapid and accurate diagnosis, for which computed tomographic pulmonary angiography (CTPA) is widely recognized as the diagnostic gold standard. However, recent advancements in imaging technologies—such as dual-energy computed tomography (DECT), photon-counting CT (PCD-CT), and artificial intelligence (AI)—offer promising enhancements to traditional diagnostic methods. This study reviews past, current and emerging technologies, focusing on their potential to optimize diagnostic accuracy, reduce contrast volumes and radiation doses, and streamline clinical workflows. DECT, with its dual-energy imaging capabilities, enhances image clarity even with lower contrast media volumes, thus reducing patient risk. Meanwhile, PCD-CT has shown potential for dose reduction and superior image resolution, particularly in challenging cases. AI-based tools further augment diagnostic speed and precision by assisting radiologists in image analysis, consequently decreasing workloads and expediting clinical decision-making. Collectively, these innovations hold promise for improved clinical management of PE, enabling not only more accurate diagnoses but also safer, more efficient patient care. Further research is necessary to fully integrate these advancements into routine clinical practice, potentially redefining diagnostic workflows for PE and enhancing patient outcomes.

## Introduction

Pulmonary embolism (PE) is a thrombotic vascular disease that can fall under venous thromboembolism (VTE) along with deep vein thrombosis [1]. Actually, this disease represents the third most common cause of cardiovascular death worldwide after stroke and heart attack [2, 3], and approximately 10 million cases occur each year worldwide [4]. In addition, during the COVID-19 pandemic, its incidence rate has increased; in fact, according to a recent meta-analysis, 16.5% of patients with coronavirus pneumonia developed PE [5–9].

**Fig. 1:**
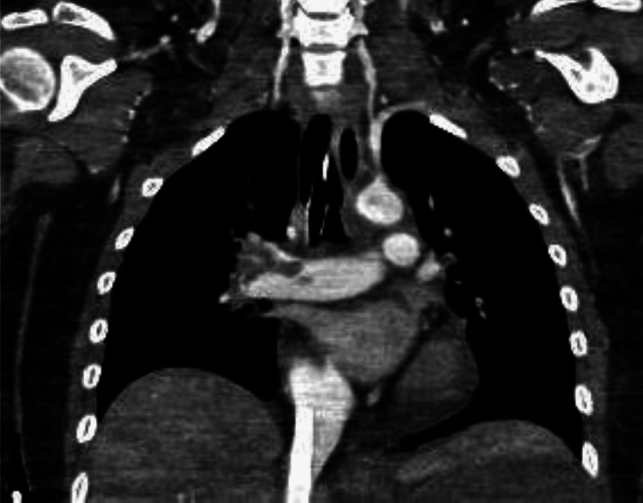
Coronal CTA scan that reveals massive arterial occlusion with failure to enhance the entire lumen due to a substantial filling defect of the right lobar arteries

**Fig. 2:**
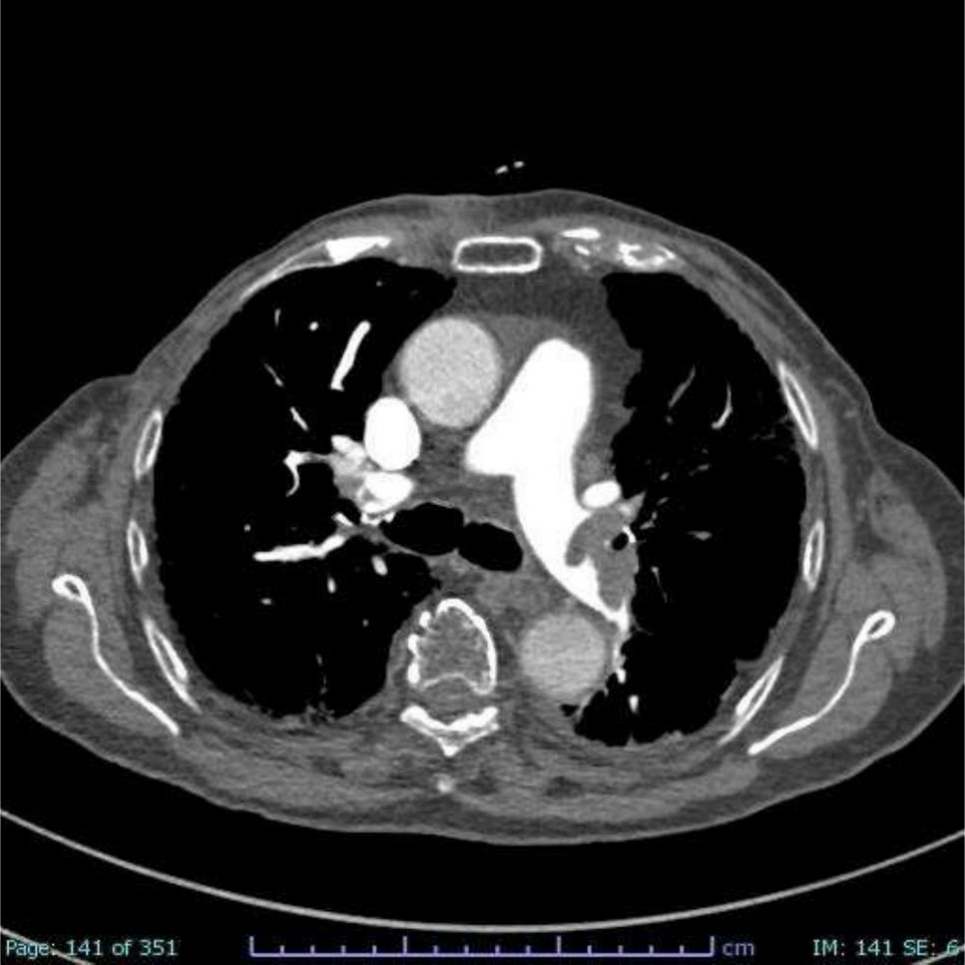
Axial CTA scan shows massive arterial occlusion with failure to enhance the entire lumen due to a substantial filling defect of both left and right lobar arteries

**Fig. 3:**
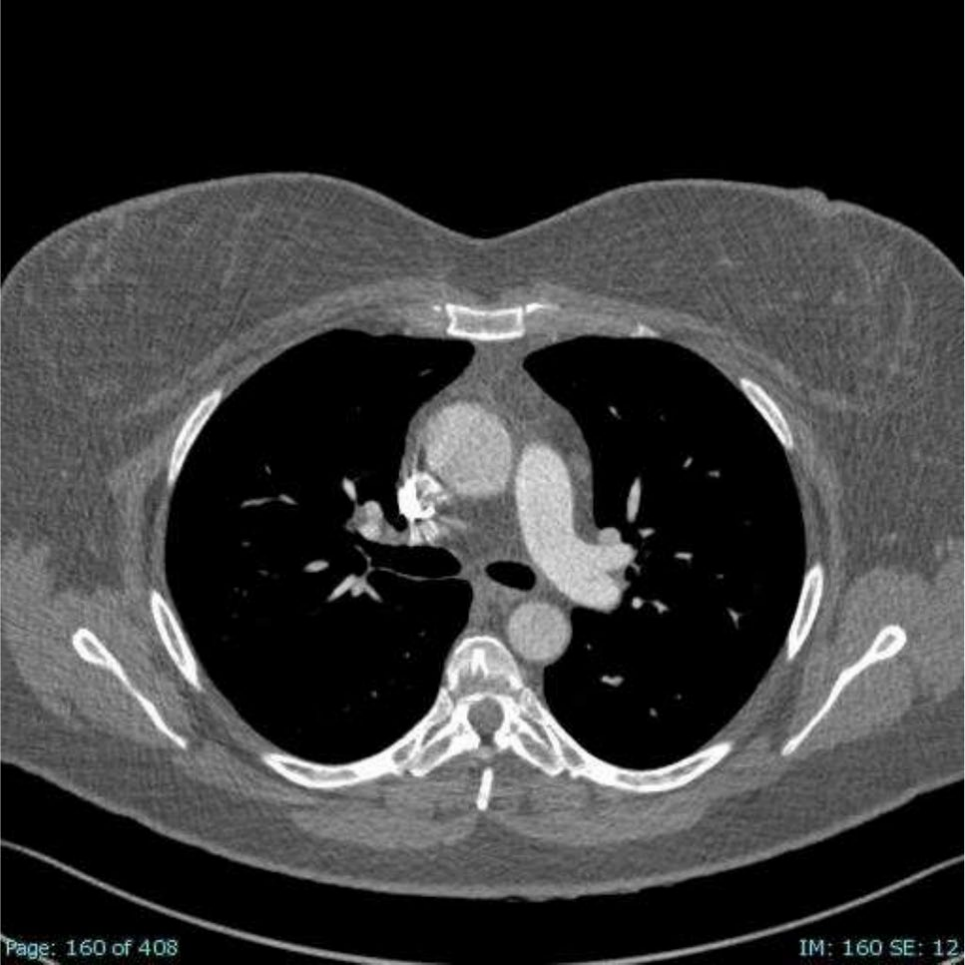
Axial CTA scan reveals submassive PE of a right subsegmental lobar artery

The etiopathogenesis of PE and VTE is due to the so-called ‘Virchow triad’, named for the physician who, in the nineteenth century, identified three factors as responsible for thrombus formation: blood flow stasis, vascular endothelial damage, and hypercoagulability [10]. Venous stasis causes an inflammatory response that exacerbates tissue damage by activating leukocytes and inflammatory factors that lead to thrombus formation in the veins [11, 12]. Several risk factors, both genetic and acquired, can increase the likelihood of developing a PE. Personal risk factors are generally permanent and include age, cancer as well as myocardial infarction and heart failure history, and coagulation disorders, such as protein C deficiency, protein S deficiency, factor V Leiden, prothrombin gene mutation and antithrombin deficiency [13, 14]. Acquired conditions related to a major risk of PE are typically transient and involve recent surgery, overall orthopedic surgery, trauma, prolonged immobility (hospitalization or long flights), and spinal cord injury [13, 14]. An alteration in coagulation, associated with an increased risk of pulmonary embolism, is also observed during pregnancy or while undergoing hormonal therapies [13, 14]. An increased risk of blood clots has also been associated with obesity, hypertension, smoking habits, hypercholesterolemia, and diabetes mellitus [13, 14]. Several studies have indicated a significant correlation between COVID-19 and an increased risk of PE [5, 7, 15]. COVID-19, particularly in severe cases, is associated with a hypercoagulable state, likely due to the combination of systemic inflammation, endothelial injury, and the activation of coagulation pathways [16]. This prothrombotic environment predisposes patients to thromboembolic events, including PE [5]. Diagnostic imaging has played a crucial role during the COVID-19 era, serving as an essential tool in the diagnosis, management, and monitoring of the disease [17–20]. Moreover, imaging has been instrumental in identifying complications of COVID-19, such as PE [6, 21, 22], acute respiratory distress syndrome [23], and secondary infections [24–26], all of which significantly impact patient outcomes. Clinical findings have shown that patients with COVID-19 who develop PE often present with more severe respiratory symptoms, and the presence of PE is associated with increased morbidity and mortality even if no correlation has been related to the COVID-19 variants yet [27, 28]. In addition to its diagnostic role, imaging has also been critical in monitoring the progression of the disease and evaluating the response to treatment, especially in patients with severe or prolonged illness. Pulmonary embolism can be classified according to clot location or hemodynamic impairment. According to the American Heart Association (AHA) and American College of Chest Physicians (ACCP) guidelines [29], PE is classified as: ‘massive’ (Fig. 1, 2) and ‘sub-massive’ (Fig. 3) based on systolic blood pressure and right ventricular (RV) dysfunction (proBNP or elevated troponin) [30, 31]. Otherwise, the updated guidelines of the European Society of Cardiology guidelines (ESC) [11] classify PE as ‘high-risk’ and ‘intermediate risk’ according to hemodynamic instability. Intermediate-risk patients can also be sub-classified into “intermediate-high” and “intermediate-low” based on PE Severity Index (PESI) score, RV dysfunction, and troponin levels [14, 32, 33]. According to both AHA and ESC, there is also a third category of PE, defined as ‘low-risk PE,’ i.e., acute PE without hemodynamic instability and right ventricular dysfunction [14, 29]. According to AHA and ACCP classification, patients prognosis is correlated to the severity of disease [34]: it is estimated that patients with massive PE have an expected mortality rate of 25 to 65%, and those with sub-massive PE have a mortality rate of 3 to 15%, while with low-risk PE patients with normal cardiac function have a mortality rate of < 1% when anticoagulant therapy is administrated [35, 36]. Clinical presentation of PE is not specific and can vary depending on the size and location of the embolism and the patient’s overall health. Dyspnea is the most common symptom and can be acute and severe in central PE or mild and transient in peripheral PE [14, 37]. Chest pain is also a common symptom of PE and is due to pleural irritation resulting from distal emboli that cause pulmonary infarction [14, 37, 38]. Syncope, cough, and hemoptysis may also occur [14, 37, 39, 40]. Extensive or central PE may present with hemodynamic instability [14, 37]. Conversely, PE can also be clinically asymptomatic and be accidentally discovered [14, 37]. Regarding the diagnosis of PE, computed tomographic pulmonary angiography (CTPA) is considered the gold standard [41–43] with a sensitivity between 96 and 100% and specificity between 96 and 100% between 89% [44]. According to ESC, CTPA should be performed (Class IC) in patients with high suspicion of PE, even if hemodynamically unstable. Furthermore, if CTPA is regular in patients with low or intermediate clinical probability, the diagnosis of PE can be excluded without further investigation (Class IA) [14]. The crucial importance of CTPA in the diagnostic and prognostic workflow of acute PE relies on both the high-grade accuracy of PE detection and on the concomitant evaluation of the pulmonary parenchyma status that may be affected by another intercurrent disease, such as lung tumors [45] or infectious disease. As a consequence, various research has been conducted on technological advancements and refinements of this crucial technique [46], also including the application of different CT modalities [dual-energy computed tomography (DECT); Photon-Counting Detector CT (PCD-CT)] finalized to an improved radiological approach. Artificial intelligence (AI) is playing a major part in this scenario, strengthening the CT arsenal with useful tools not only aimed at the achievement of safer examinations, with dose and/or contrast dose reductions, but also concerning an increase of diagnostic performance with computer-aided diagnosis [47].

Our aim is then to highlight the importance of CTPA in the diagnosis, follow-up, and evaluation of PE and to shed light on new technologies and perspectives that currently are or have future potential to improve the overall management of this condition.

## Imaging techniques

### Past and current imaging

To achieve a more comprehensive review of the literature, diagnostic techniques no longer commonly used in clinical practice were included, as they nonetheless represented important milestones in the diagnosis of pulmonary embolism prior to the advent of CTPA.

#### Chest radiography

Chest radiography represents the first radiological tool employed in patients with dyspnea, allowing the diagnosis of clinical conditions such as pneumonia or pulmonary edema [48]. However, in PE, chest radiography does not offer enough details for a precise diagnosis [30] and is recommended as initial imaging in suspected PE only in pregnant patients [49].

All over the years, several research studies have identified some radiological findings in PE patients with high specificity but with low sensitivity: in 1938, Westermark described for the first time a difference between embolism with and without infarction on an X-ray examination. When an area of infarct is present, it appears as a wedge-shaped, dense, homogeneous shadow, whereas in cases of PE without infarction, ischemia occurs in the branches of the pulmonary artery on the distal side of the embolus and manifests as a clarified area with reduced vascular markings that correspond to the territory supplied by the affected artery. Despite this, vascularization remains intact in the central regions of the lung. However, the vascular structures abruptly terminate at the boundary of the area of non-vascularization [50]. The area of PE with infarction was better described in 1940 by Hampton and Castelman as a wedge-shaped opacity that contacts the pleural surface and has a sharply convex medial border [51]. In the late 1950 s, Fleischner described another sign of PE in cases of massive unilateral PE: a prominent central pulmonary artery is observed due to diverting the pulmonary arterial blood to the unobstructed lung [52]. In 1962, he also delineated some ancillary findings, such as the well-known “Fleischner line”, defined as plate-like atelectasis secondary to inhibited respiratory excursion [48, 53, 54].

#### Ventilation/perfusion (V/Q) lung scintigraphy

Another imaging technique that should be mentioned, which is not specific to the detection of PE since its introduction in the mid-1960s, is V/Q lung scintigraphy [48]. Limitations related to V/Q lung scans include a high rate of nondiagnostic results and the inability to provide an alternative diagnosis [55, 56]. Although V/Q mismatched defects are not always diagnostic for PE, a V/Q lung scan is usually appropriate as initial imaging in high pretest probability and in low or intermediate pretest probability with a positive D-dimer [49]. Moreover, V/Q lung scintigraphy could find its utility as a preliminary study in young women with a low to intermediate pretest clinical probability of PE or in patients allergic to intravenous contrast medium or with renal failure [48, 57]. V/Q imaging has also been recommended to monitor patients with positive PE to assess for recurrence or evaluate their response to treatment [48]. V–P scintigraphy in pregnant women with suspected PE is accepted as first-line imaging and is also appropriate as a second-line choice if the chest X-ray is normal [57–59]. V/Q findings suggestive of PE have a positive predictive value of less than 10% and include (listed in increasing frequency order): one to three small segmental perfusion defects, matched V-P abnormalities in two or three zones of one lung, triple-matched defects in the upper or middle lung zone, the “stripe sign”, perfusion defects smaller than the corresponding regions of increased opacity on radiography, nonsegmental perfusion abnormalities [55, 60, 61]. V/Q with single photon emission computed tomography (SPECT) allows tomographic images with higher contrast resolution than V/Q scintigraphy and avoids the limit of overlapping small perfusion defects by normal tissue [55, 56]. Indeed, V/Q SPECT has been demonstrated to have a high negative predictive value and a low indeterminate rate [56].

#### Angiography

The role of conventional angiography is usually not appropriate for diagnosing PE due to the lower overall accuracy compared to CTPA [49]. According to the literature, the limitations of angiography are related to the difficult assessment of subsegmental clots, vessel overlap, and cardiac or respiratory motion [48].

#### Magnetic resonance imaging (MRI)

A limited application in the detection of PE is currently reserved also for MRI. According to the American College of Radiology (ACR), Appropriateness Criteria® MR angiography pulmonary arteries without and with intravenous contrast may be appropriate as initial imaging in suspected PE both at low or intermediate pretest probability with a positive D-dimer and at high pretest probability [49, 62]. In 2010 a multicenter prospective study (PIOPED III) demonstrated the higher sensitivity in PE diagnosis of the combination of MR angiography and venography rather than MR angiography alone, when both are technically well performed [63]. To address the limitations caused by respiratory and cardiac motion artifacts, MRI is conducted using gradient-recalled echo imaging techniques during breath-holding [60]. Similarly to CTA, in MRI studies, PE consists of a partial filling defect with the opacification absence of the artery distal to the clot and a sudden drop in vessel capacity [48, 64]. Other pulmonary artery signs are the “railway sign” on the longitudinal axis of a vessel and the “polo mint” on the perpendicular axis of a vessel [60]. MRI can also demonstrate parenchymal findings commonly shown in PE, such as wedge-shaped pleura opacities that can evolve into scars, cavities, irregular peripheral linear opacities, peripheral nodules, and bands [60, 64].

#### Compression ultrasonography (CUS)

A cheaper and faster imaging technique is the CUS duplex doppler of the lower extremity, which may be appropriate as initial imaging in high pretest probability PE. However, not all authors agree with this statement [31] and suggest reserving it for patients with risk factors or symptoms of deep vein thrombosis (DVT) [65]. Both patients with a history of previous or chronic DVT or with suspected acute DVT could be studied with Color Doppler flow imaging that has been demonstrated to be complementary to compression ultrasonography [66]. Certainly, the US of the lower extremities is usually useful for pregnant patients with suspected PE [49]. Thrombus can have different gray-scale echogenicity but, commonly, it appears anechoic during the acute phase and increases its echogenicity during the time [48, 67] and, at Color-Doppler flow evaluation, the presence of the clot is visible as an intraluminal filling defect [48, 66]. Overall, according to the ACR, the presence of DVT does not confirm PE, but it does increase its likelihood. Conversely, a negative extremity ultrasound study does not rule out PE, although it significantly reduces its likelihood [49]. A common and critical error is to cease further investigation after a normal CUS, but this should not happen because the absence of proximal DVT does not rule out PE, and additional thoracic imaging is required unless an alternative diagnosis is evident or PE is no longer suspected [55]. Pros of US duplex Doppler include the detection of both non-vascular and vascular abnormalities such as Baker cysts, aneurysms, and pseudoaneurysms, whereas cons are related to the operator-dependent nature of the technique and to patient physical limits; moreover, Asymptomatic proximal lower extremity or isolated symptomatic calf vein thrombosis cannot be detected with good sensitivity [48].

#### Positron emission tomography (PET)

The utility of PET in detecting pulmonary embolism PE has been investigated, highlighting its potential advantages. Gallium-68 V/Q PET/CT offers lower radiation exposure, improved resolution, and shorter acquisition times compared to SPECT, making it particularly beneficial for cancer patients where CTPA fails to provide definitive or conclusive results for suspected acute PE [68, 69]. Furthermore, PET serves as a feasible alternative in patients with contraindications to contrast agents used in CTPA. Additionally, 18F-labelled fluoro-2-deoxyglucose PET has revealed two distinguishable signs of PE: the “rim sign,” associated with pulmonary infarction, and abnormal cardiac uptake, indicating right heart strain [70]. Particularly in oncology patients, where PET/CT is increasingly utilized for disease diagnosis and staging, the presence of focal or curvilinear FDG uptake may raise suspicion for PE, prompting further diagnostic evaluation with CTPA [71]. Given the promising results, studies involving a larger patient population are needed to confirm its validity in the oncological population.

#### Computed tomographic pulmonary angiography (CTPA)

Over the years, the previously mentioned techniques have found their role in the diagnosis of PE, but nowadays, the gold standard is represented by CTPA. In 2006 the Prospective Investigation of Pulmonary Embolism Diagnosis II (PIOPED II) trial demonstrated that multidetector CT angiography (CTA)—CT venography (CTV) has higher sensitivity for diagnosis of PE compared to CTA alone, with comparable specificity and either CTA or CTA-CTV, have high predictive accuracy when supported by a consistent clinical assessment [44]. In 2022 the ACR Appropriateness Criteria® stated that the CTPA should be the initial imaging tool in low or intermediate pretest probability with a positive D-dimer as well as in high pretest probability and in pregnant women with suspected PE [49]. At CTA, PE appears as an intraluminal filling defect, and the thrombus may completely obstruct the lumen, sometimes causing the enlargement of the vessel, or it may partially obstruct blood flow in a central or eccentric location [48, 60]. The central location produces the “railway track” sign on imaging parallel to the long axis of a vessel and the “polo mint” sign on perpendicular images, whereas the peripheral location is observed as an acute angled intraluminal filling defect [72]. Ancillary findings not specific to acute PE are represented by peripheral wedge-shaped areas of hyperattenuation that may outline infarcts and linear bands [48, 60, 72]. Secondary to PE, right ventricular failure could happen, and despite sometimes being stable and normotensive at presentation, patients are at a higher risk of PE-related mortality [73]. CTPA findings related to right ventricular dysfunction consist of right ventricular dilatation, where the right ventricular (RV) cavity is wider than the left ventricular (LV) cavity in the short axis (RV/LV ratio > 0.9), with or without contrast material reflux into the hepatic veins, shift of the interventricular septum towards the left ventricle, azygos vein dilatation, and a PE index exceeding 60 [48, 72–74]. Chronic PE is shown at CTPA as the following findings: complete occlusion of a vessel smaller than the nearby open vessels, a peripheral, crescent-shaped intraluminal defect forming obtuse angles with the vessel wall, contrast material passing through thickened, often narrower arteries due to recanalization, a web or flap within a contrast-filled artery [72, 75]. Less frequent findings include a mosaic perfusion pattern, numerous bronchial or other systemic collateral vessels, and calcification within asymmetrical vessel thickening [72, 75, 76]. Chronic PE ancillary findings at CTPA consist of changes secondary to pulmonary hypertension, like increased diameter of the pulmonary artery (greater than 33 mm) [72, 77] and the presence of pericardial fluid [72, 78]. CTPA can play a crucial role in detecting differential diagnoses in cases with clinical symptoms similar to those of PE without embolism at imaging. Indeed, pericarditis can be observed as a pericardial thickening or fluid; in aortic dissection, an endoluminal flap is recognized; in acute myocardial infarction, imaging consists of a filling defect in a coronary artery or as a perfusion defect of the myocardium [72]. Other mimics can involve pulmonary parenchyma, such as pneumonia, lung cancer, pleural disease, or esophagus, such as esophageal rupture or esophagitis, or chest wall, such as rib fractures [72]. Imaging modalities, particularly CXR and computed CT scans, have been pivotal in detecting characteristic lung abnormalities associated with COVID-19, such as ground-glass opacities, consolidation, and the “crazy-paving” pattern [20]. These findings have helped in the early identification of severe cases, guiding treatment decisions and patient triage, especially when testing resources were limited or results were delayed [79–82]. The CTPA technique has evolved over time to address issues related to radiation exposure and reduce the use of intravenous contrast agents. The following chapters describe new protocols, advanced acquisition techniques such as DECT and PCD-CT, and the role of AI.

##### CTPA technical optimization: dose and contrast reduction

Chest X-ray has historically had a role in the diagnosis of PE with specific radiographic findings (e.g., Westermark sign) [83–86]; it has been replaced by CTPA, which is considered the imaging gold standard to confirm or rule out suspected PE. As such, it is a commonly performed diagnostic examination that requires a relatively high radiation exposure to achieve its results in terms of specificity and sensitivity. Radiation exposure is related to an increased lifetime cancer risk and gametal damage, particularly for younger and female patients [87]. For this reason, in the last two decades, different techniques for dose reduction have been implemented to minimize radiation exposure while maintaining adequate image quality, in line with the “as low as reasonably achievable” (ALARA) principle [88]. Among the different possible approaches, a reduction of tube voltage peak (kVp) and scan time, modulation of tube current (mA), the use of high pitch scanning or the scan range customization (e.g. in pregnant patients) or iterative reconstruction (IR) are the most frequently employed [89–92]. The decrease of X-ray tube potential leads to a reduction of radiation dose and volume of contrast, despite increased image noise and consequently a decrease of imaging quality (particularly in overweight patients) [93]. Nevertheless, several studies demonstrate that a decrease in tube potential from 120 to 100kVp may lead to a reduction of up to 30% in radiation dose without significantly impacting the image quality [94]. For instance, Szucs-Farkas et al. compared two CTPA protocols: one with a tube voltage of 120 kV and 100 mL of contrast volume and another with 100 kV and 50 mL of contrast; the results did not demonstrate statistically significant differences in terms of diagnostic accuracy in the group with lower tube voltage and volume contrast compared to the classic protocol [95]. Moreover, even with voltages of 80 kV, CTPA’s protocols were deemed viable for the diagnosis of PE for normal weights; in obese patients, the protocol was theoretically feasible but would require high-power tubes with excellent anti-scattering systems with elevated entrance dose, making the application of the protocol difficult to safely implement on this cohort of patients [96, 97]. IR is an advanced image reconstruction technique used in CT imaging that, differently from traditional reconstruction methods, such as filtered back projection (FBP) which reconstructs images directly from raw data, iteratively refines an initial image estimate to better match the acquired data, reducing imaging noise and artifacts [98], a high-quality image reconstruction featured by DL systems can be beneficial for a more precise final diagnosis. Kaul et al. demonstrated that a combination of a statistical iterative reconstruction (ASIR) and a lower tube potential can reduce dose radiation with an imaging quality sufficient for diagnosing PE. The study affirms that a protocol with a tube potential of 120 kV and the use of FBP does not show significant differences in the qualitative and quantitative analysis compared to a protocol with a tube potential of 100 kV and the use of an IR, so in this way, the effective dose is 54.4% lower in the protocol with 100 kV than to 120 kV [99]. Another study by Lu et al. demonstrated that a high-pitch 80-kV CTPA, when combined with IR, provided diagnostically comparable images to those of a standard-pitch 100-kVp pulmonary CTA with FBP but at half the radiation dose [100]. Scanning in a high pitch can reduce oversampling, mitigate or completely avoid motion artifacts (an important feature when examining patients with shortness of breath or chest pain who have difficulty holding their breath), and decrease image acquisition time, resulting in reduced radiation dose and volume of contrast [101, 102]. A recent study by Schönfeld et al. explored the viability of a CTPA protocol with ultra-fast high-pitch mode (pitch: 3.2), ultra-low contrast volume administration (20 mL), with kVp and tube current adaptation in normal weight patients, in comparison with a standard CTPA. The results showed that subjective image quality was similar between the two protocols, while objective image quality was slightly lower in the high-pitch CTPA group but still in acceptable ranges for making a diagnosis. So, the CTPA protocol with low contrast dose and high pitch scan in normal-weight patients rendered diagnostic images for the detection of PE and concomitantly reduced radiation exposure compared to a standard CTPA [103]. These applications (summarized in Table 1) are often used in combination, partly included in the everyday clinical routine, and partly still in development. They can provide the radiologist with an additional weapon to control and adjust contrast volume and radiation exposure, making CT scans safer but still effective.

**Table 1:** Literature about the methods of dose and contrast reduction in the detection of PE

Author	Year	Type of study	Number of CTPA	Performance score
Kaul et al. [99]	2014	Retrospective	44	Overall diagnosability = 4.54 ± 0.45
Lu et al. [100]	2014	Prospective	100	Sensitivity = 93.3% [95% CI: 68.1–99.8] Specificity = 97.1% [95% CI: 85.1–99.9] PPV = 93.3% [95% CI: 68.1–99.8] NPV = 97.1% [95% CI: 85.1–99.9]
Szucs-Farkas et al. [95]	2011	Retrospective	3350	Sensitivity = 81.3% [95% CI: 78–84]; Specificity = 98.2% [95% CI: 98–99]; PPV = 44.4 [95% CI: 33–59] NPV = 0.19 [95% CI: 0.16–0.22]

### New CT technologies

In recent years, different innovations in CT technology have developed, intending to increase imaging quality (making diagnosis more accurate) and, at the same time, reduce impairing CT image defects (movement/breathing artifacts, image noise). As previously shown, these innovations also cut radiation exposure and the use of high contrast volume. Among these CT innovations, the most studied technologies include DECT and PCD-CT. DECT offers the possibility to provide both morphological and functional pulmonary information of the lung in a single contrast-enhanced examination through different post-processing techniques (like linear blending, iodine maps, and virtual monoenergetic (VM) reconstructions), which have been shown to improve image quality and visualization of vascular structures [46]. In fact, DECT allows for simultaneously depicting filling occluding defects of the pulmonary arteries (PA) and resultant perfusion alterations in the lung parenchyma [104–106]. It has been demonstrated that the DECT technique can improve the imaging quality even with a small amount of contrast agent. In fact, the use of monoenergetic image reconstructions improves vessel attenuation and contrast-to-noise ratio, allowing for a reduction in the amount of deployed iodine contrast [107]. The development of third-generation DECT, characterized by advanced dual-energy voltage combinations, thicker and thinner filtration systems, and enhanced spectral separation of X-rays, partially addresses the limitations associated with the high radiation exposure inherent to first-generation DECT systems [108].

PCD-CT is an innovative imaging technology that utilizes photon-counting detectors instead of conventional energy-integrating detectors used in traditional CT scanners [109, 110]. With PC-CT, the specific energy level of every individual incoming photon is measured and directly converted to an electrical sign. This leads to different advantages, such as the suppression of electronic noise below predefined thresholds, higher spatial resolution with preserved dose efficiency, and efficient mitigation of metal-induced artifacts [111, 112]. In 2023 Pannenbecker et al. published a study to assess the image quality of a high-pitch PCD-CTPA scan protocol with ultra-low contrast medium and radiation dose for the diagnosis of acute PE and compare its performance to an established DE-CTPA protocol on a third-generation dual-source CT (DSCT) scanner. The work demonstrates that the acquisition of CTPAs on a PCD-CT scanner with 25 mL of iodinated contrast medium maintains high imaging quality and that the reduction of effective radiation dose by 51.2% (1.4 mSv vs. 3.3 mSv in the group with DSCT) was associated with no significant loss of subjective image quality or diagnostic confidence. For this reason, the CTPA with PCD allows lower contrast volume and radiation dose reduction compared with a third-generation DSCT with an electronic detector [113]. DECT and PDCT are not regarded as routine diagnostic procedures, especially considering their rather high production and maintenance cost (especially for PCD-CT). However, due to their excellent research results, their clinical use will potentially increase during the next years.

## Artificial intelligence

Decoding CTPA to detect PE is laborious and mentally challenging for radiologists, given the extensive number of CT images and vessels that necessitate evaluation. Radiologists must meticulously track down to the sub-branch of the sixth stage of the pulmonary artery or even smaller for potential abnormalities [114, 115]. In addition, an accurate diagnosis could be hindered by respiratory movement artifacts, flow artifacts, partial volumetric effects, presence of lymph nodes, blood vessel bifurcation, and, consequently, the interpretation process is prone to inaccuracies, leading to delayed diagnoses. Artificial Intelligence-based systems can function as a secondary reader, promptly interpreting and accelerating the diagnosis of PE [116–120]. Several methods based on quantitative image analysis, known as CAD, have been proposed for the detection of PE in CTPA scans. An optimal CAD system should enhance the efficiency of a radiologist’s workflow by emphasizing regions with potential clinical significance, allowing for increased focus on these specific areas [47, 121]. Overall, Özkan et al. [121] set up a CAD system that proved to be highly beneficial as a supplementary tool for radiologists, serving effectively as a second reader, and their method demonstrates superior performance in detecting PE compared to those documented in the previous existing studies without this approach. In the era of AI, there is a pressing need for models that reduce the substantial workload of radiologists and minimize inter-observer variability in large-scale early disease Chest-X Ray (CXR) screening. For instance, Sun et al. proposed a CAD model that significantly improves the performance of CXR multi-label classification and enhances the interpretability of label correlations, thereby providing novel insights and methodologies for automated clinical diagnosis [122]. Deep learning (DL) is one of the most proficient applications of artificial intelligence, able to “learn” image features of high-quality data with the assistance of convoluted neural networks (CNN). DL has been extensively employed in chest imaging, in the evaluation and follow-up of both oncological and infectious diseases for pulmonary [123–132] and cardio-pulmonary [133–137] districts. Li et al. [115] proposed a DL algorithm able to achieve a positive correlation between arterial grade and detection rate of the emboli; moreover, among the participants, a good sensitivity of 90.9% and an average false positive of 2.0 were obtained. For the first time, in 2014, Lahiji et al. [47] retrospectively compared the use of IR with FBP in a prototype of PE CAD. According to their results, implementation of the hybrid IR algorithm resulted in a notable and continuous reduction in false-positive annotations generated by PE CAD when compared to those made by radiologists on FBP reconstructions. Recently, some deep-learning-based algorithms have been proposed in the literature to retrospectively train and test the detection of acute PE in CTPA, like those provided by Grenier et al. [138] and Langius‐Wiffen et al. [139]. Although both obtained high accuracy rates in detecting suspected acute PEs, the first has the limits of not comparing algorithm results with reports of radiologists. Both previously cited studies do not integrate clinical data in their algorithm. Conversely, clinical data were analyzed by Liu et al. [140]. The DL-CNN assessed by the Chinese study group demonstrated the correlation between clot burden of acute PE and clinical scores (Qanadli and Mastora scores) and with parameters of right ventricular function on CTPA (such as RVa/LVa, RVd/LVd, the septal angle) [140]. The Basel study group also correlated the detection of acute PE in CTPA to communication times and patient management in the Emergency Department for the first time [114]. They confirm the feasibility of DL-assisted PE detection with high diagnostic accuracy; however, the mere integration of these tools into the radiology department is still insufficient to yield significant effects on clinical performance measures. According to the pathophysiology mechanisms, it is crucial to promptly and appropriately diagnose and manage Incidental Pulmonary Emboli (IPEs) identified during routine chest CT scans with contrast, even when conducted for reasons unrelated to thromboembolic disease. Chest CT examinations with contrast enhancement may employ diverse techniques when not conducted following a CTPA protocol. Specifically, the timing of the contrast medium bolus varies and is not usually optimized for achieving ideal opacification of the pulmonary artery. Topff et al. [141], prospectively applying commercially available AI tools on routine CT scans of a cohort of oncology patients, demonstrated exceptional diagnostic accuracy and considerably reduced the time to diagnosis, particularly in a context marked by a backlog of examinations. In addition, a retrospective study conducted by Batra et al. [142] demonstrated that a commercially available AI tool demonstrated a 99.8% Negative Predictive Value (NPV) and an 86.8% Positive Predictive Value (PPV) in detecting PE on conventional contrast-enhanced chest CT examinations of cancer patients. In conclusion, a proficient AI system should establish an optimal operating threshold, finding a balance between sensitivity and specificity. This equilibrium enables the acceleration of the diagnostic workflow without overwhelming radiologists with false positive cases, as a high number of such cases can lead to alarm fatigue. Even in the case of chronic PE, a machine learning system trained on CT features developed by Gawlitza et al. has proven useful in non-invasive risk stratification and, consequently, in the proper management of the patient [143]. Chronic pulmonary embolism is a promising area for the application of AI in the near future. In fact, a recent systematic review highlighted that there is still limited literature regarding AI models tested and validated for the diagnosis of chronic PE [144]. Specifically, the review noted an overall reasonable reporting of AI model design and training, but inconsistent reporting of the datasets used, which limits their transparency [144].

In the context of PE detection, it becomes evident that a deep learning system can effectively function as a secondary reader, providing immediate interpretation and prioritization of positive studies. Ultimately, an AI-based tool holds the potential to speed up PE diagnosis. Given the critical importance of timely diagnosis, the integration of a triage model can significantly enhance the overall quality of care of the patient. Table 2 provides an overview of the literature on AI applications in identifying PE.

**Table 2:** Literature about AI’s application in PE detection

Author	Year	Type of study		Number of CTPA	Performance score
Topff et al. [141]	2023	Prospective, Single center	11,736		Sensitivity: 91.6% [95% CI: 86.7, 95.8]; Specificity: 99.7% [95% CI: 99.6, 99.8]; PPV: 80.9% [95% CI: 74.7, 86.4]; NPV: 99.9% [95% CI: 99.8, 99.9]; Accuracy: 99.6% [95% CI: 99.5, 99.7]
Langius‐Wiffen et al. [139]	2023	Retrospective, single center	3316		Sensitivity: 96.8% [95% CI: 95.5–98.1]; specificity: 99.9% [95% CI: 99.8–100]; PPV: 99.7 [95% CI: 99.3–100.0]; NPV: 99.1 [95% CI: 98.8–99.5]
Grenier et al. [138]	2023	Retrospective, multicenter	109		Sensitivity: 91.4% [95% CI: 86.4–95.0%]; specificity: 91.5% [95% CI: 86.8–95.0%]; accuracy: 91.5%
Batra et al. [142]	2022	Retrospective, single center	3016		Sensitivity: 82.5 [95% CI: 67.2–92.7]; Specificity 99.8 [95% CI: 99.5–99.9]; PPV: 86.8 [95% CI: 73.1–94.1] NPV: 99.8 [95% CI: 99.5–99.9]
Schmuelling et al. [114]	2021	Retrospective, single center	1808		Sensitivity 79.6% (95% CI: 70.8–87.2%); specificity 95.0% (95% CI: 92.0–97.1%); PPV 82.2% (95% CI: 73.9–88.3); NPV 94.1% (95% CI: 91.4–96%)
Li et al. [115]	2021	Retrospective, single center	85		Sensitivity: 90.9%; average number of false positives per case: 2.0
Liu et al. [140]	2020	Retrospective, single center	590		Sensitivity: 94.6%; Specificity: 76.5% AUC: 0.926 (95% CI: 0.884–0.968)
Lahiji et al. [47]	2014	Retrospective, Single center	66		Sensitivity 70.3% for FBP; Specificity 30.3% for FBP; Accuracy 77.3%
Özkan et al. [121]	2013	Retrospective, Single center	33		Sensitivity of 95.1%

## Future challenges

Prospective multicenter studies are essential to validate the efficacy of emerging technologies such as DECT, PDCT, and AI algorithms for the automated detection of PE. These investigations should aim to enhance radiologist performance and improve patient clinical outcomes. Moreover, future studies should include data such as comorbidities and laboratory results to improve the performance of AI tools. In addition, future research should investigate the impact of timely identification of incidental pulmonary emboli on both morbidity and mortality. Another interesting future application could be the introduction of radiomics in the workflow of PE imaging. Applications in the field of vascular pathology have already been studied in other districts for the density classification of thrombotic and embolic findings [145–149] but may provide thriving options in the management and PE evaluation as well [150], especially in the preliminary risk assessment process. Additionally, the future of PE radiological management revolves around further standardizing and homogenizing the reporting portion. Several studies explored the possibility of creating structured report schemes in radiology [151–163] to increase the speed of reporting and provide clinicians with easy, standardized, and more practical insight into the patient’s radiological status to better choose the following therapeutic scheme.

## Conclusions

Pulmonary embolism represents an extremely urgent condition that requires a precise and detailed diagnosis. Technological progress has ensured the use of increasingly high-performance diagnostic tools both in the acquisition and post-processing phases. CTPA is the main diagnostic tool, and thanks to the optimization techniques that reduce contrast and radiation dose, it is a safe approach for the patient. In addition, DECT and PDCT are not employed during routine diagnostic procedures, but they have proven to be highly accurate methods in PE diagnosis. AI approaches could improve the diagnostic workflow either in risk assessment or to assess PE during CT studies obtained with no dedicated protocol to increase PPV in cancer patients.
